# Supplementary material for: Dynamic Inverse Relationship Between Cell-Free DNA and Anti-dsDNA Antibodies in Experimental SLE Highlights the Potential for Targeted Immunomodulatory Therapy
Source: Pathophysiology. 2025 Sep 16;32(3):48. doi: 10.3390/pathophysiology32030048 (PMC12452575; doi:10.3390/pathophysiology32030048)
Supplement: Supplementary file 1 [file pathophysiology-32-00048-s001.zip › pathophysiology-3604246-supplementary.pdf]

Supplementary materials

Article

Dynamic Inverse Relationship Between Cell-Free DNA and Anti-dsDNA Antibodies in Experimental SLE Highlights the Potential for Targeted Immunomodulatory Therapy

Mark M. Melamud [1], Evgeny A. Ermakov [1,2], Anna S. Tolmacheva [1], Georgy A. Nevinsky [1,2] and Valentina N. Buneva [1,2,*]

[1] Institute of Chemical Biology and Fundamental Medicine, Siberian Branch of the Russian Academy of Sciences, 630090 Novosibirsk, Russia
[2] Department of Natural Sciences, Novosibirsk State University, 630090 Novosibirsk, Russia
* Correspondence: buneva@niboch.nsc.ru

Table S1. Dynamics of changes in cfDNA and anti-dsDNA Abs concentrations within the groups between time points.

Time point 1	Time point 2	Dynamics	P-value
CfDNA dynamics, experimental group			
Week -2	Week 8	↓	P=0.61
Week 8	Week 14	↑	**P=0.025**
Week 14	Week 22	↓	P=0.15
Week 22	Week 28	↓	P=0.5
Week 28	Week 36	↓	P=0.22
CfDNA dynamics, control group			
Week -2	Week 8	↑	P=0.44
Week 8	Week 14	↑	P=0.06
Week 14	Week 22	↑	P=0.11
Week 22	Week 28	↓	**P=0.014**
Week 28	Week 36	↑	P=1
Anti-dsDNA Abs dynamics, experimental group			
Week -2	Week 8	↑	**P=0.0025**
Week 8	Week 14	↓	**P=0.0054**
Week 14	Week 22	↑	P=1
Week 22	Week 28	↑	**P=0.0025**
Week 28	Week 36	↓	**P=0.0086**
Anti-dsDNA Abs dynamics, control group			
Week -2	Week 8	↓	**P=0.014**
Week 8	Week 14	↑	P=0.29
Week 14	Week 22	↑	**P=0.04**
Week 22	Week 28	↑	**P=0.014**
Week 28	Week 36	↑	P=0.08

Statistical significance of differences was assessed using the Wilcoxon test.
